# Hepatitis C Elimination: Opportunities and Challenges in 2023

**DOI:** 10.3390/v15071413

**Published:** 2023-06-22

**Authors:** Gadeer Taha, Levy Ezra, Naim Abu-Freha

**Affiliations:** 1 Department of Gastroenterology, Rambam Health Care Campus, Haifa 31096, Israel; 2 Medical School for International Health, Ben-Gurion University of the Negev, Beer-Sheva 84101, Israel; 3 Institute of Gastroenterology and Hepatology, Soroka University Medical Center, Beer-Sheva 84101, Israel; 4 Faculty of Health Sciences, Ben Gurion University of the Negev, Beer-Sheva 84105, Israel

**Keywords:** hepatitis C, elimination, barriers, health system, provider, patients

## Abstract

Hepatitis C Virus (HCV) infection is a leading etiology of liver cirrhosis and its associated complications, namely, decompensated cirrhosis. As such, hepatitis C potentially necessitates liver transplantation and may result in death. Recently, HCV treatment has evolved. Current HCV treatment is effective in curing HCV; some of the agents are pan-genotypic. Numerous countries have adopted an initiative to eliminate HCV. Achieving elimination poses many challenges; it requires improved availability and accessibility of pan-genotypic therapy. Barriers exist at the level of the collective healthcare system and at the level of the individual healthcare providers and patients. Therefore, organized national and local efforts are needed. Surmounting these barriers calls for interventions concerning screening, linkage to care, and treatment delivery. Pertinent barriers include inadequate availability of screening, ill-equipped laboratory testing before treatment, and insufficient access to treatment. Interventions should seek to decentralize laboratory testing and treatment provision, increase funding for resources and personnel, and spread awareness. Special consideration should be allocated to at-risk populations, such as intravenous drug users, refugees, and prisoners. Computerized medical filing and telemedicine have the potential to refine HCV management by enhancing detection, availability, accessibility, and cost-effectiveness.

## 1. Introduction

Hepatitis C virus (HCV) persists as a serious health problem in many regions of the world. The prevalence of HCV has fallen dramatically since oral direct-acting antivirals (DAA) were introduced. However, according to the World Health Organization (WHO), the global prevalence of chronic HCV infection remains as high as 58 million, including 3.2 million cases in adolescents and children [1]. In addition, there are approximately 1.5 million new infections per year [1]. WHO estimated that in 2019, 290,000 people died of hepatitis C. Mortality was mostly a result of complication of the chronic disease with cirrhosis and hepatocellular carcinoma (HCC) [1]. Hepatitis C ranges in severity from a mild, acute illness to a long-term, chronic condition that can lead to serious liver complications, such as cirrhosis and liver cancer. Many people with hepatitis C do not have noticeable symptoms, especially in the early stages. Therefore, it is difficult to detect the infection without testing. During the last decade, diagnosis and treatment have been simplified. The old process included a lot of laboratory testing during the treatment, treatment with significant side effects, and a low rate of sustained virologic response (SVR) [1,2,3,4].

Direct-acting antiviral medications cure the infection in most cases, and the treatment with oral DAAs is highly effective with minimal adverse effects [2,3,4]. Chronic HCV infection involves a long-term inflammatory process. The inflammation leads to fibrosis, cirrhosis, and other complications. Treatment decreases the virus’s inflammatory process, which, in turn, diminishes the severity of fibrosis and the rate of complications. Various studies demonstrate improving the fibrosis grade following treatment with DAA. Among patients with a fibrosis grade of F3 (using transient elastography values between 9.6 and 14.6 kPa), fibrosis regressed in 58% of the patients, remained stable in 30% of the patients, and progressed in 12.5% of patients treated with DAA [5]. Mild fibrosis regressed shortly after treatment; however, regression was delayed in patients with advanced fibrosis at baseline [6]. Fibrosis regression decreases complications, such as cirrhosis development, HCC, and the need for transplantation. Several studies reported a reduction in the rate of HCC following DAA treatment [5,7,8,9,10]. The goal of the present review is to highlight and provide an update regarding opportunities and challenges in the elimination of hepatitis C. The challenges should be managed by the countries, Health Maintenance Organizations (HMO), and health policymakers. The barriers and interventions relate to three different levels: the health system level, the provider level, and the patient level. In the present review, the barriers were highlighted. These barriers and interventions are discussed based on published experiences from different centers, countries, and HMOs. Issues related to models for the healthcare of HCV, the cascade of care, and the cost-effectiveness of HCV screening and treatment were mentioned as well.

## 2. World Health Organization (WHO) Initiative

The WHO has initiated several global efforts to address the public health burden of hepatitis C. The WHO’s initiative to eliminate HCV was proposed in 2016. The strategy sets targets for reducing new hepatitis infections, increasing access to testing and treatment, and improving surveillance and monitoring. It focuses on promoting equity, engaging communities, and integrating hepatitis services into existing health systems. The stated objective includes reducing the incidence and mortality. The target is a 90% reduction in chronic HCV incidence and a 65% reduction in HCV mortality by 2030 [11]. Many countries adopted this goal, and established HCV elimination programs. However, the SARS-CoV-2 pandemic’s detrimental impact on the health system slowed or even suspended HCV elimination programs. HCV testing and treatment fell, which increased morbidity and mortality [12,13,14,15].

## 3. Access and Barriers

**Figure 1:**
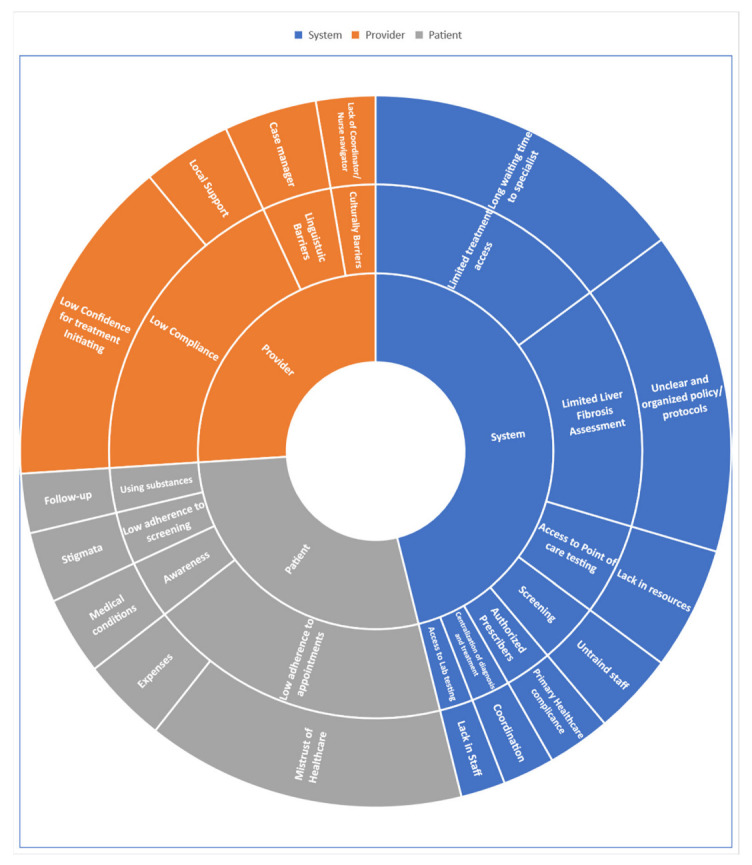
Barriers and Challenges for HCV elimination.

While there have been significant advances in the treatment of hepatitis C, many people still do not have access to treatment. Lack of access is due to various barriers, such as high costs, limited healthcare resources, and lack of awareness. Barriers exist at multiple levels, from the provider and patient to the overarching system. The various barriers to HCV elimination at different levels of healthcare provision are detailed in the following sections and summarized in Figure 1.

## 4. Actions Required

The rate of new infections, morbidity, and mortality of chronic HCV infections represent a significant burden for health systems. First, intervention is needed to prevent hepatitis C. Effective prevention strategies can be implemented through harm reduction measures, and safe injection practices reduce the transmission of hepatitis C among high-risk populations, such as people who inject drugs (PWIDs). With the advent of highly effective antiviral medications, interventions are needed to screen at-risk patients and treat diagnosed cases of HCV. Intervention should emphasize the accessibility and availability of screening, linkage to care, treatment, and follow-up. Interventions for HCV elimination at the different levels of the healthcare system are summarized in Table 1 and discussed in detail.

## 5. System-Level Barriers

Health systems need to overcome barriers in the preparation and the initiation of HCV treatment. The system-level barriers are summarized in Figure 1. The health system structure varies by country. In most countries, the health ministry regulates the health system. Meanwhile, in other countries, the health ministry provides health services. Programs should be suited to the form of the healthcare organization which they are intended to function within. The primary barrier is access to HCV screening to identify undiagnosed, infected people. HCV antibody testing should be available according to the regional prevalence of high-risk people or the general population. The following at-risk populations should be invited for HCV antibody testing: intravenous drug users, pre-1992 blood transfusion recipients, pre-1992 organ transplant recipients, pre-1987 clotting factor concentrate recipients, infants born to HCV viremic mothers, hemodialysis patients, HIV-positive cases, men who have sex with men, and migrants from a country with high HCV prevalence [16,17,18]. Special considerations are necessary for PWIDs and prisoners [2,16,17,18].

A high rate of hepatitis C in a country is defined as a ≥2% or ≥5% HCV antibody seroprevalence in the general population [19]. In such a case, universal population screening is necessary. New point-of-care oral anti-HCV testing [20,21,22,23] is a viable option for universal screening in regions with high rates of HCV. The advantages of point-of-care oral anti-HCV testing are simple administration, swift turnaround, and coherent results. Another barrier is the lack of computerized medical files. Computerized health systems should be utilized to locate people with risk factors. Health systems that do not computerize data should offer opportunistic testing at visits to general practitioners.

Lack of health system funds for non-invasive fibrosis assessment modalities, such as laboratory-based assessment, AST to Platelet Ration Index (APRI), FIB-4 score (calculation of age, AST, ALT, and platelets), Fibrotest (calculation of α2-macroglobulin, haptoglobin, apolipoprotein A1, and bilirubin) and gamma-glutamyl transpeptidase (GGT), or FibroScan (liver elastography and liver stiffness measurement) tests to detect liver disease severity, in addition to long wait times and large geographic distances to see HCV specialists, are complex barriers to treatment initiation [24,25,26].

Overall, health systems should adopt strategies to increase testing with community-based hepatitis testing services and provide access to treatment for all grades of fibrosis. In some countries, including Russia, access to treatment is restricted to advanced fibrosis stages [26]. Lack of a healthcare policy regarding HCV treatment, deficient funding, and poor access to treatment are substantial barriers that must be conquered to reach the elimination of HCV. Another barrier is initiating HCV treatment through a decentralized process in order to minimize bureaucracy and paperwork. A previous case study demonstrated the effectiveness and feasibility of decentralized HCV testing and treatment for high-risk patients in primary healthcare settings [27]. Authorization of prescribers is critical. In most countries, a prescription for HCV treatment must be given by a specialist (hepatologist, gastroenterologist or infection disease specialist). In contrast, there are countries in which HCV treatment may be prescribed by a primary healthcare provider or pharmacist [27,28,29].

A health system intervention should address all three levels of HCV healthcare cascade: screening, linkage to treatment, and treatment (Table 1). National programs should be planned and executed according to the quantity and quality of national resources available. It is the health system’s responsibility to outline policies and regulations that support hepatitis C elimination efforts, specifically, increased funding for hepatitis C programs.

## 6. Provider-Level Barriers

The role of direct health service providers is of the utmost importance as the provider is the first to meet the HCV patients. The practitioner plays a crucial role in starting and following the entire process. The process starts with screening, continues to diagnosis and treatment, and proceeds with follow-up. Family doctors may prescribe DAA to treat HCV patients with a low fibrosis grade [2] and forego unnecessary consultations with gastroenterologists or hepatologists, thus expediting the time from diagnosis to treatment. Nevertheless, there are several barriers at the level of family doctors. These barriers are summarized in Figure 1. For instance, most family doctors are overbooked with symptomatic patients and other assignments. Moreover, low adherence to treatment, substance abuse, and lack of treatment for substance abusers impede the diagnosis and treatment of HCV patients [30,31].

Perhaps some family doctors do not believe that the treatment of hepatitis is their responsibility. During the period of interferon treatment, a significant minority of addiction medicine physicians were providing HCV antiviral treatment [32]. Unfamiliarity with DAA treatment for HCV patients may affect confidence in initiating treatment. Local clinics lack support for HCV treatment, which requires staff for blood draws, case managers or links to care coordinators, and an authoritative protocol for HCV testing [24]. Medical workers and resources for contacting patients to begin HCV testing and treatment are needed. In health systems with good quality family medicine, the family doctor and the local clinic’s staff should be a part of the screening and treatment of HCV. In health systems where other models are used for hepatitis C diagnosis and treatment, such as mobile clinics or multidisciplinary centers, other facilities, or treatment in the outpatient’s clinic of hospitals, the family doctor does not play a major role in the process.

## 7. Patient-Level Barriers

The principal barrier at the patient level is awareness. Specifically, a lack of awareness about the importance of screening and the consequences of untreated chronic HCV infection hinders treatment initiation. The barriers at the patient level are presented in Figure 1. HCV awareness campaigns at local and national levels can address the issue of awareness. Campaigns must be culturally and linguistically adapted to each country, particularly for minorities with high rates of HCV infection. Psychosocial barriers persist as well, such as failure to keep appointments and obtain testing, loss of follow-up, and substance abuse [33]. Widespread stigmas, mistrust of healthcare, lack of a symptomatic condition, fear of diagnostic testing, and fear of adverse effects deter patients [24]. The stigma associated with hepatitis C due to its association with drug injection or other high-risk behaviors deters individuals from getting screened. Fear of discrimination, social isolation, or negative judgment prevent people from using hepatitis C screening services. Furthermore, access to screening and healthcare services are limited by geographic location—transportation and financial constraints—and insurance coverage [24,28,29,30,31,32,33].

In addition, certain patients are difficult to contact. Most notably, impoverished minorities, PWIDs, and the homeless population are not easy to reach. Medical expenses are a significant barrier. Expenses include direct and indirect costs, payments for medication and payments for secondary items such as travel costs. In situations where resources are scarce, HCV treatment should be rationed. Patients with dire prognoses may not qualify as candidates for treatment. For example, patients with short life expectancies, advanced malignancy, and other severe diseases without advanced liver fibrosis [33]. Language and cultural barriers prevent individuals from seeking hepatitis C screening [24]. Language barriers can impede communication between patients and healthcare providers. Difficulties navigating through mistrust of the healthcare system are additional barriers [24]. Cultural beliefs, practices, and misconceptions about hepatitis C affect individuals’ perceptions and decisions about screening [33]. Interventions are necessary at the patient level to overcome the aforementioned obstacles. Interventions must be designed and carried out by the health ministry, health organizations, and third-sector organizations. Intervention should be performed at local (such as clinics) high, and national levels. Public awareness campaigns, educational programs, and community outreach efforts regarding hepatitis C and its risks encourage screening and treatment. Multidisciplinary centers are an excellent option. In multidisciplinary centers, patients can receive specialized care to address all of their medical and extra-medical needs. It is important to note that adherence to screening and treatment is a key part of the process. Low adherence results in process interruption and failure to reach SVR [33]. Interventions at the patient level are summarized in Table 1.

## 8. Telemedicine

Telemedicine using phone calls, video conferencing, remote monitoring, and mobile apps allows healthcare providers to consult, diagnose, and treat patients remotely. Telemedicine has become increasingly popular as a result of technological advances, the need to improve access to healthcare, and the SARS-CoV-2 pandemic. Telemedicine emerged as an essential component of healthcare during the SARS-CoV-2 pandemic and was adopted by many countries [34,35,36]. Telemedicine is used for different medical services, such as chronic disease management, mental health counselling, and urgent care consultations [34,35,36]. Telemedicine is beneficial for chronic HCV patients because HCV requires laboratory testing and fibrosis assessment before treatment begins. The prerequisite assessment can be conducted via telemedicine remotely. Subsequently, according to the severity of the disease, patients could be invited for in-person visits to the clinic or treated without face-to-face meetings. Telemedicine allows patients to receive timely medical care by bypassing in-person visits. As such, telemedicine reduces wait times for treatment initiation, reduces healthcare costs, and improves patient outcomes. To be sure, telemedicine is not suitable for all patients and medical conditions. Only appropriate patients should be selected.

In sum, there are several uses of telemedicine among HCV patients: remote consultations from specialists or nurse navigators for diagnosis and preparing the patient for treatment (laboratory testing and fibrosis severity assessment). In a virtual meeting, a treatment decision can be made. However, patients suffering from advanced liver disease at primary assessment should visit a specialist in person. Telemedicine saves time and other costs, and HCV patients are generally highly satisfied with telemedicine experiences. Satisfaction was found to be equivalent to in-person encounters [37]. In certain settings, such as prison, telemedicine consultations among HCV patients is more efficient than typical clinical practice. It saves money while preserving effectiveness and satisfaction [38].

In telemedical practice, the case manager and other staff should be organized with clearly defined roles. In institutions where family doctors are the case managers, telemedicine could be used for physicians to communicate with HCV patients. Virtual specialist consultation and computerized medical files help in patients’ assessment prior to treatment. Telemedicine is not feasible in certain settings. Elderly people may have difficulty using new technology [39]. Issues regarding regulatory and legal factors and reimbursements hinder telemedicine implementation [40]. Telemedicine is very limited in a health system without digital medical records. Paper charting makes it difficult to uncover information regarding comorbidity and medications. In some regions, cultural and linguistic barriers make telemedicine difficult to use. Cultural sensitivity and translations are viable solutions to these problems.

## 9. Models for HCV Healthcare

Different models of healthcare exist for intervention, screening, investigation, and the treatment of patients with HCV. Generally, the objective of the models is to increase uptake of and adherence to HCV treatment. Models are developed according to the characteristics of the population served and their health system. Several models have also been suggested based on local experience. National versus local and general population versus high-risk population questions are priority issues. The next step is enrolling people in the intervention program for laboratory testing, treatment of the viremic patients, and follow-up [41].

In specific settings, integrating the treatment of HCV into existing centers, such as those serving PWIDs, is an effective strategy. In these centers, a relationship and trust have been previously cultivated. However, in most countries, a novel public health approach is needed. Establishing specialized multidisciplinary centers is recommended for countries with high HCV prevalence. Public health programs conducted by the health ministries and HOMs suffice as well. The indispensable parts of any initiative are screening and linkage to treatment. A mobile, community-based, adequately funded program is suitable for remote regions [42]. Regardless of the model chosen for screening and linkage to care, it will entail additional manpower, facilities, and resources.

## 10. Cascade of Care

The cascade of care for HCV diagnosis and treatment process is an important framework for evaluating services, access, and program effectiveness at the population level. It can track the WHO’s goal of eliminating hepatitis C by the year 2030. Reaching this goal requires real-time evaluation and improvement of the process in time.

The cascade of care for HCV was investigated in several studies. The cascade of care is subdivided into different stages: HCV antibody testing, HCV RNA testing, genotype testing, treatment initiation, and achieving SVR [43]. In this study, data were collected between the years 1999 and 2018. It is important to mention that nowadays, in the era of pan-genotypic treatment, the genotype testing stage is irrelevant. Mendlowitz et al. showed that among 4962 patients, 83% of those with positive HCV antibodies were tested for HCV RNA and 60% of them were positive and 1002 (40%) initiated treatment. In another study from Ontario, less than half of patients with positive RNA test results initiated treatment; people in older age groups were more likely to perform HCV RNA testing and initiate treatment. Other studies also showed different rates of HCV RNA. A study from a mass screening and treatment campaign in Rwanda between 2017 and 2019 showed 57% positive RNA among people with positive HCV antibodies, 52% initiated treatment, and 72% completed treatment [42], while another study from Korea showed a low performance of RNA testing of 36.2%, with 72.9% RNA positivity, and a treatment initiation rate of 57.6% [44], and SVR (89.7%, 88%) [42,44]. The significant discrepancy in the RNA positivity of participants in the studies mentioned above most probably lies in the different settings. One study included participants in mass screening of the general population, while the other study was performed in a single tertiary hospital with positive HCV antibodies.

Defining and evaluating the gaps and barriers in real time in the care cascade is imperative for effective interventions. Improving adherence among positive HCV RNA patients, increasing the treatment initiation rates, and overcoming barriers to the prioritization of HCV elimination demands constant monitoring to ensure that programs at local and national levels allocate the resources and efforts to screening and linkage to care for chronic hepatitis C patients. In addition, quality measurement indicators should be defined. These indicators should include data that could be followed and compared in different time periods; these quality measurement indicators could include, but are not limited to, the number of new patients screened, the rate of HCV RNA testing after a positive HCV antibodies result, the rate of treatment initiation, and the rate of SVR. In addition, indicators could be defined for people diagnosed with HCV but still not treated.

## 11. Cost Effectiveness of HCV Screening and Elimination

An analysis of HCV screening’s cost effectiveness was performed in different countries. The models were tested in diverse setting and in countries with varying prevalence of HCV. Numerous strategies were tried. Analysis showed significant benefits of screening in terms of its impact on morbidity or mortality in the local settings of different countries. Detection and treatment of HCV patients diminished the HCV burden [45,46,47,48,49]. The economic benefit from a long-term perspective encourages policymakers to implement screening and treatment programs for HCV patients. An elimination strategy needs to be tailored to each country. Tactics vary depending on local prevalence and population characteristics, ensuring cost effectiveness for the target population with either general or high-risk screening. In each country, according to the local data, the cost-effectiveness analysis should be calculated according to the local strategy. Local data should determine the local strategy, varying from screening an entire population to screening specific high-risk patients, such as PWIDs, specific age groups, minorities, refugees, prisoners, or others.

## 12. Discussion

In the era of highly effective DAA treatment, active interventions have the potential to eliminate HCV as stated by the WHO’s initiative (90% reduction in chronic HCV incidence and 65% reduction in HCV mortality by 2030). The goal can be achieved by reducing transmission, morbidity, and mortality. Screening for early detection and treatment of HCV is essential to reach elimination. The action and interventions should be carried out at multiple levels: health systems, providers, and patients. Most barriers to achieving elimination are related to the health system. Interventions to overcome these barriers will improve the availability and accessibility of screening, assessment before treatment, and treatment of chronic HCV. National programs should be planned in countries with high rates of HCV while accounting for the available human and systemic resources. The interventions should target all HCV population groups by modeling data to predict people with increased risk for HCV and screening them. Increasing awareness regarding the importance of HCV screening and treatment of HCV through education encourages people to use the intervention once it is offered. Training healthcare providers and increasing availability and accessibility of testing and treatment contribute substantially to elimination. The implementation of appropriate models for screening and treatment reduces chronic HCV infection and increases the number of people screened and treated. Egypt is exemplary, with a high prevalence of HCV, 10% seroprevalence, and a 7% viremic rate among the population aged 15–59 years [50]. An effective model for screening and treatment was implemented. The Egyptian National Committee for Control of Viral Hepatitis provided free DAA HCV treatment. The infrastructure of the national program was an important aspect of its success. It included a wide network of specialized centers [51]. These specialized centers provided integrated care for HCV patients. The offered comprehensive care: screening, evaluation, treatment, and follow-up. The care was provided by a well-trained multidisciplinary team [52]. These centers numbered 64 in 2018, with <50 km between each center around the country [51]. More than two million patients were treated by 2018 (about 40% of all patients with HCV), with an SVR in more than 90% of those treated as a consequence of Egypt’s organized national program. It achieved high accessibility and availability of screening, evaluation, and treatment of HCV [53]. Another outstanding accomplishment of Egypt’s national program was its participation rate. About 49.6 million (79.4%) people from the 62.5 million target population participated in screening between 1 October 2018, and 30 April 2019 [53]. On the other hand, South Korea, which has a low prevalence of HCV, has yet to implement a national hepatitis C elimination program. A recent study from Korea showed an RNA testing rate of 36.2% among 3253 people with positive HCV antibodies [44].

Overall, many factors interact to determine the success of an elimination program. These factors include national policy plan, availability of epidemiological data, awareness of HCV among the general population and high-risk groups, screening programs, prevention programs, treatment guidelines, publicly funded diagnostic testing for HCV, and treatment for all patients. Additionally, electronic medical records and telemedicine are critical aspects affecting programs for HCV elimination. Telemedicine makes specialty consultation available and expedites patient preparation for treatment. However, telemedicine and the infrastructure for telemedicine are available only in some health care systems; telemedicine will not be an option for HCV treatment in less-developed health systems. The health system should prioritize at-risk populations, such as refugees, PWIDs, and prisoners. Eradicating HCV will decrease cirrhosis, HCC, and the need for liver transplantation, making intervention cost effective despite the high cost of DAA [54,55,56]. Focusing on prisoners, prison custody officers (PCOs) are additional players that have a potential role in promoting the treatment of hepatitis C, safeguarding, stigma, confidentiality, and education are important issues related to the PCOs that help in service delivery and treatment in prisons [57].

Lastly, promoting international collaboration and coordination among countries, organizations, and stakeholders to share best practices, resources, and expertise in hepatitis C elimination efforts is pivotal. This involves partnerships with global health organizations, governments, civil society, and other stakeholders to leverage collective efforts. It is important to note that the strategies and actions needed for hepatitis C elimination vary depending on the local epidemiology, health system capacity, and socio-economic status of each country or region. A comprehensive approach that addresses prevention, testing, treatment, health systems strengthening, advocacy, community engagement, surveillance, and global collaboration is crucial to eliminate hepatitis C as a public health threat [58]. Additionally, the hepatitis fund accelerates viral hepatitis elimination; an international hepatitis fund was established for hepatitis elimination support. This fund aims to help resource-limited countries afford program funding and technical assistance, and particularly non-governmental organizations, research organizations, or clinics, which can develop an effective intervention for hepatitis elimination [58].

## 13. Conclusions

The elimination of chronic HCV is urgent and important. To reach this goal, clever interventions by health systems to overcome barriers are needed. Resources, training, and awareness are important factors for success. Despite the challenges entailed, effort to eliminate hepatitis C is essential to reduce morbidity and mortality. Overall, countries that performed mass screening and treatment campaigns for HCV as a part of national programs had extraordinary success, while countries that did not carry out any national program remain with a heavy HCV burden.

## Figures and Tables

**Table 1:** The interventions needed at the system, provider, and patient level.

	System-Level	Provider Level	Patients Level
Screening	Screening high-risk patients (PWID, prisoners, and others)	Proactive action: patient invitation for screening	Stopping substance abuse
	Modeling high-risk patient screening according to medical files	Being aware with cultural and linguistic barriers	Keeping appointments
	Universal screening in countries with high HCV prevalence		Adherence to screening
Linkage to care	Improving HCV antibody and viral load testing accessibility and availability	Preparing patients for treatment (laboratory test including antibody and HCV viral load)	Compliance with preparation before treatment, and laboratory testing
	Availability of the point-of-care/self-testing according to the local needs	Case and resource management at the local level	Insurance coverage
	Availability of testing for non-invasive liver disease severity assessment (FIB-4, APRI, Fibrotest, FibroScan)	Using doctor-to-doctor virtual consultation if available	
	Decentralization of the patient’s investigation/ patient’s preparation for treatment		
Treatment	Universal access to treatment	Be convinced treatment is possible, easy, and simple	Paying medical expenses
	Shortening wait time for specialist consultation	Appropriate education for HCV treatment initiation, in regions where providers are authorized to do so	Adherence to treatment
	Using telemedicine throughout all phases		
	Decentralization of treatment approval and prescriptions		
	Adding prescribers (internist, family doctors, or pharmacists) according to the local needs		
	Clear healthcare policy/national program/protocols		
	Special consideration for people who inject drugs, refugees, and prisoners		
Other elements	Education and training of the providers	Increasing knowledge, education Staff familiarity with the process of screening, investigation and treatment	Continuing follow-up
	Increasing awareness among the population regarding the importance of the screening and treatment	Using telemedicine when possible	
	Other manpower Nurse navigator		

## Data Availability

No additional data are available.

## References

[1] https://www.who.int/news-room/fact-sheets/detail/hepatitis-c.

[2] Abu-Freha N., Jacob B.M., Elhoashla A., Afawi Z., Abu-Hammad T., Elsana F., Paz S., Etzion O. (2022). Chronic hepatitis C: Diagnosis and treatment made easy. Eur. J. Gen. Pract..

[3] EASL (2020). EASL recommendations on treatment of hepatitis C final update of the series. J. Hepatol..

[4] Ghany M.G., Morgan T.R. (2020). AASLD-IDSA Hepatitis C Guidance Panel. Hepatitis C guidance 2019 update: American association for the study of liver diseases–infectious diseases society of America recommendations for testing, managing, and treating hepatitis C virus infection. Hepatology.

[5] Shiha G., Soliman R., Hassan A.A., Mikhail N.N.H. (2022). Changes in hepatic fibrosis and incidence of HCC following direct-acting antiviral treatment of F3 chronic hepatitis c patients: A prospective observational study. Hepatoma Res..

[6] Zakareya T., Elhelbawy M., Elzohry H., Eltabbakh M., Deif M., Abbasy M. (2021). Long-Term Impact of Hepatitis C Virus Eradication on Liver Stiffness in Egyptian Patients. Can. J. Gastroenterol. Hepatol..

[7] Kanwal F., Kramer J., Asch S.M., Chayanupatkul M., Cao Y., El-Serag H.B. (2017). Risk of hepatocellular cancer in HCV patients treated with direct-acting antiviral agents. Gastroenterology.

[8] Li D.K., Ren Y., Fierer D.S., Rutledge S., Shaikh O.S., Lo Re V., Simon T., Abou Samra A.-B., Chung R.T., Butt A.A. (2018). The short-term incidence of hepatocellular carcinoma is not increased after hepatitis C treatment with direct-acting antivirals: An Archives study. Hepatology.

[9] Ioannou G.N., Green P.K., Berry K. (2017). HCV eradication induced by direct acting antiviral agents reduces the risk of hepatocellular carcinoma. J. Hepatol..

[10] Shiha G., Mousa N., Soliman R., Nnh Mikhail N., Adel Elbasiony M., Khattab M. (2020). Incidence of HCC in chronic hepatitis C patients with advanced hepatic fibrosis who achieved SVR following DAAs: A prospective study. J. Viral Hepat..

[11] World Health Organization Global Health Sector Strategy on Viral Hepatitis 2016–2021. https://apps.who.int/iris/bitstream/handle/10665/246177/WHO-HIV-2016.06-eng.pdf;jsessionid=60A93ADD1A191FF6A0FA823314D24C43?sequence=1.

[12] Yeo Y.H., Gao X., Wang J., Li Q., Su X., Geng Y., Huang R., Wu C., Ji F., Sundaram V. (2022). The impact of COVID-19 on the cascade of care of HCV in the US and China. Ann. Hepatol..

[13] Blach S., Kondili L.A., Aghemo A., Cai Z., Dugan E., Estes C., Gamkrelidze I., Ma S., Pawlotsky J.M., Razavi-Shearer D. (2021). Impact of COVID-19 on global HCV elimination efforts. J. Hepatol..

[14] Hoenigl M., Abramovitz D., Flores Ortega R.E., Martin N.K., Reau N. (2022). Sustained Impact of the Coronavirus Disease 2019 Pandemic on Hepatitis C Virus Treatment Initiations in the United States. Clin. Infect. Dis..

[15] Kondili L.A., Buti M., Riveiro-Barciela M., Maticic M., Negro F., Berg T., Craxì A. (2022). Impact of the COVID-19 pandemic on hepatitis B and C elimination: An EASL survey. JHEP Rep..

[16] World Health Organization (2017). Guidelines for the Screening, Care and Treatment of Persons with Hepatitis C Infection.

[17] Ferraro M., Painter M. (2019). Hepatitis C—Screening, diagnosis, management & treatment. Osteopath. Fam. Phys..

[18] Guss D., Sherigar J., Rosen P., Mohanty S.R. (2018). Diagnosis and Management of Hepatitis C Infection in Primary Care Settings. J. Gen. Intern. Med..

[19] WHO Recommendation. https://apps.who.int/iris/bitstream/handle/10665/254621/9789241549981-eng.pdf?sequence=1.

[20] Liu L., Zhang M., Hang L., Kong F., Yan H., Zhang Y., Feng X., Gao Y., Wang C., Ma H. (2020). Evaluation of a new point-of-care oral anti-HCV test for screening of hepatitis C virus infection. Virol. J..

[21] Lee S.R., Yearwood G.D., Guillon G.B., Kurtz L.A., Fischl M., Friel T., Berne C.T., Kardos K.W. (2010). Evaluation of a rapid, point-of-care test device for the diagnosis of hepatitis C infection. J. Clin. Virol..

[22] Cha Y.J., Park Q., Kang E.-S., Yoo B.C., Park K.U., Kim J.-W., Hwang Y.-S., Kim M.H. (2013). Performance evaluation of the OraQuick hepatitis C virus rapid antibody test. Ann. Lab. Med..

[23] Pallarés C., Carvalho-Gomes A., Hontangas V., Conde I., Di Maira T., Aguilera V., Benlloch S., Berenguer M., López-Labrador F.X. (2018). Performance of the OraQuick hepatitis C virus antibody test in oral fluid and fingerstick blood before and after treatment-induced viral clearance. J. Clin. Virol..

[24] Litwin A.H., Drolet M., Nwankwo C., Torrens M., Kastelic A., Walcher S., Somaini L., Mulvihill E., Ertl J., Grebely J. (2019). Perceived barriers related to testing, management and treatment of HCV infection among physicians prescribing opioid agonist therapy: The C-SCOPE Study. J. Viral Hepatol..

[25] Cardoso A.C., Figueiredo-Mendes C., Villela-Nogueira C.A., Marcellin P. (2022). Staging Fibrosis in Chronic Viral Hepatitis. Viruses.

[26] Isakov V., Nikityuk D. (2022). Elimination of HCV in Russia: Barriers and Perspective. Viruses.

[27] Shilton S., Markby J., Sem X., Siva S., Zain R., Menetrey C., Yuswan F., Nasir N., Andrieux-Meyer I., -Easterbrook P. (2021). Feasibility, effectiveness, and lessons learned from the introduction of decentralised HCV testing and treatment at primary healthcare clinics in three regions in Malaysia. J. Hepatol..

[28] Mandel E., Underwood K., Capraru C., Shah H., Janssen H., Feld J., Biondi M. (2021). Working towards elimination by understanding province to province variability in the HCV cascade of care in Canada. Hepatology.

[29] Patel A., Wang S., Gallipani A., Brogden R., Xu B., Adedeji M., Yango J. (2021). Partnership for elimination: Ambulatory care clinical pharmacists dismantling barriers to hepatitis C cure. Hepatology.

[30] Grebely J., Tyndall M.W. (2011). Management of HCV and HIV infection among people who inject drugs. Curr. Opin. HIV AIDS.

[31] Myles A., Mugford G.J., Zhao J., Krahn M., Wang P.P. (2011). Physicians’ attitudes and practice toward treating injection drug users with hepatitis C: Results from a national specialist survey in Canada. Can. J. Gastroenterol..

[32] Litwin A.H., Kunins H.V., Berg K.M., Federman A.D., Heavner K.K., Gourevitch M.N., Arnsten J.H. (2007). Hepatitis C management by addiction medicine physicians: Results from a national survey. J. Subst. Abus. Treat..

[33] Malespin M., Harris C., Kanar O., Jackman K., Smotherman C., Johnston A., Ferm J., de Melo S.W., Scolapio J.S., Nelson D.R. (2019). Barriers to treatment of chronic hepatitis C with direct acting antivirals in an urban clinic. Ann. Hepatol..

[34] Omboni S., Padwal R.S., Alessa T., Benczúr B., Green B.B., Hubbard I., Kario K., Khan N.A., Konradi A., Logan A.G. (2022). The worldwide impact of telemedicine during COVID-19: Current evidence and recommendations for the future. Connect Health.

[35] Solari-Twadell P.A., Flinter M., Rambur B., Renda S., Witwer S., Vanhook P., Poghosyan L. (2022). The impact of the COVID-19 pandemic on the future of telehealth in primary care. Nurs. Outlook.

[36] Reicher S., Sela T., Toren O. (2021). Using Telemedicine During the COVID-19 Pandemic: Attitudes of Adult Health Care Consumers in Israel. Front. Public Health.

[37] Talal A.H., Sofikitou E.M., Wang K., Dickerson S., Jaanimägi U., Markatou M. (2023). High Satisfaction with Patient-Centered Telemedicine for Hepatitis C Virus Delivered to Substance Users: A Mixed-Methods Study. Telemed. e-Health.

[38] Mao A., Tam L., Xu A., Osborn K., Sheffrin M., Gould C., Schillinger E., Martin M., Mesias M. (2022). Barriers to Telemedicine Video Visits for Older Adults in Independent Living Facilities: Mixed Methods Cross-sectional Needs Assessment. JMIR Aging.

[39] Cuadrado A., Cobo C., Mateo M., Blasco A.J., Cabezas J., Llerena S., Fortea J.I., Lázaro P., Crespo J. (2021). Telemedicine efficiently improves access to hepatitis C management to achieve HCV elimination in the penitentiary setting. Int. J. Drug Policy.

[40] Gajarawala S.N., Pelkowski J.N. (2021). Telehealth Benefits and Barriers. J. Nurse Pract..

[41] Bobb R., Malayala S.V., Ajayi E., Wimbush A., Bobb A. (2023). Hepatitis C Treatment in Persons Who Inject Drugs in a Medication Assisted Treatment Program: A Retrospective Review of an Integrated Model. J. Prim. Care Community Health.

[42] Nisingizwe M.P., Makuza J.D., Janjua N.Z., Bansback N., Hedt-Gauthier B., Serumondo J., Remera E., Law M.R. (2023). The Cascade of Care for Hepatitis C Treatment in Rwanda: A Retrospective Cohort Study of the 2017–2019 Mass Screening and Treatment Campaign. Viruses.

[43] Mendlowitz A.B., Bremner K.E., Krahn M., Walker J.D., Wong W.W.L., Sander B., Jones L., Isaranuwatchai W., Feld J.J. (2023). Characterizing the cascade of care for hepatitis C virus infection among Status First Nations peoples in Ontario: A retrospective cohort study. CMAJ.

[44] Yoo S.H., Kim M., Kim S., Lee J.I., Lee K.S., Lee H.W., Lim J.H. (2023). The care cascade for hepatitis C virus and prognosis of chronic hepatitis C patients treated with antiviral agents in a tertiary hospital. BMC Gastroenterol..

[45] Opstaele L., Bielen R., Bourgeois S., Moreno C., Nevens F., Robaeys G., Robaeys G., van Vlierberghe H. (2019). Who to screen for hepatitis C? A cost-effectiveness study in Belgium of comprehensive hepatitis C screening in four target groups. Acta Gastro Enterol. Belgica.

[46] Krauth C., Rossol S., Ortsäter G., Kautz A., Krüger K., Herder B., Stahmeyer J.T. (2019). Elimination of hepatitis C virus in Germany: Modelling the cost-effectiveness of HCV screening strategies. BMC Infect. Dis..

[47] Gountas I., Sypsa V., Papatheodoridis G., Souliotis K., Athanasakis K., Razavi H., Hatzakis A. (2019). Economic evaluation of the hepatitis C elimination strategy in Greece in the era of affordable direct-acting antivirals. World J. Gastroenterol..

[48] Lim A.G., Scott N., Walker J.G., Hamid S., Hellard M., Vickerman P. (2021). Health and economic benefits of achieving hepatitis C virus elimination in Pakistan: A modelling study and economic analysis. PLoS Med..

[49] Scott N., Kuschel C., Pedrana A., Schroeder S., Howell J., Thompson A., Wilson D.P., Hellard M. (2020). A model of the economic benefits of global hepatitis C elimination: An investment case. Lancet Gastroenterol. Hepatol..

[50] Ministry of Health and Population, El-Zanaty and Associates (2015). Egypt Health Issues Survey 2015.

[51] Omran D., Alboraie M., Zayed R.A., Wifi M.N., Naguib M., Eltabbakh M., Abdellah M., Sherief A.F., Maklad S., Eldemellawy H.H. (2018). Towards hepatitis C virus elimination: Egyptian experience, achievements and limitations. World J. Gastroenterol..

[52] El-Akel W., El-Sayed M.H., El Kassas M., El-Serafy M., Khairy M., Elsaeed K., Kabil K., Hassany M., Shawky A., Yosry A. (2017). National treatment programme of hepatitis C in Egypt: Hepatitis C virus model of care. J. Viral Hepatol..

[53] Waked I., Esmat G., Elsharkawy A., El-Serafy M., Abdel-Razek W., Ghalab R., Elshishiney G., Salah A., Abdel Megid S., Kabil K. (2020). Screening and Treatment Program to Eliminate Hepatitis C in Egypt. N. Engl. J. Med..

[54] Tatar M., Keeshin S.W., Mailliard M., Wilson F.A. (2020). Cost-effectiveness of Universal and Targeted Hepatitis C Virus Screening in the United States. JAMA Netw. Open.

[55] Chhatwal J., Kanwal F., Roberts M.S., Dunn M.A. (2015). Cost-effectiveness and budget impact of hepatitis C virus treatment with sofosbuvir and ledipasvir in the United States. Ann. Intern. Med..

[56] Scott D.N., Palmer M.A., Tidhar M.T., Stoove P.M., Sacks-Davis D.R.S., Doyle A.J.S., Pedrana D.A.J., Thompson P.A., Wilson P.D.P., Hellard P.M. (2021). Assessment of the cost-effectiveness of Australia’s risk-sharing agreement for direct-acting antiviral treatments for hepatitis C: A modelling study. Lancet Reg. Health West. Pac..

[57] Jack K., Thomson B., Patterson A. (2015). PTU-102 “you can’t treat ‘em ‘till you get the security right”: Prison officers views about hepatitis C testing and treatment in prisons. Gut.

[58] Thornton J. (2023). Hepatitis Fund aims to accelerate viral hepatitis elimination. Lancet.

